# Prediction of small-for-gestational-age neonates at 33–39 weeks’ gestation in China: logistic regression modeling of the contributions of second- and third-trimester ultrasound data and maternal factors

**DOI:** 10.1186/s12884-022-04991-7

**Published:** 2022-08-25

**Authors:** Danping Xu, Xiuzhen Shen, Heqin Guan, Yiyang Zhu, Minchan Yan, Xiafang Wu

**Affiliations:** 1 Reproductive Center, Taizhou Hospital of Zhejiang Province, Wezhou Medical University, Wenzhou, China; 2 Department of Obstetrics and Gynecology, Taizhou Hospital of Zhejiang Province, Wezhou Medical University, Wenzhou, China; 3 Department of Ultrasonic Diagnosis, Taizhou Hospital of Zhejiang Province, Wezhou Medical University, Wenzhou, China

**Keywords:** Small-for-gestational-age, Ultrasound, Estimated fetal weight, Abdomen circumference, Third-trimester screening

## Abstract

**Objectives:**

A screening model for prediction of small-for-gestational-age (SGA) neonates (SGAp) was established by logistic regression using ultrasound data and maternal factors (MF). We aimed to evaluate the ability of SGAp as well as abdominal circumference (AC) and estimated fetal weight (EFW) measurements to predict SGA neonates at 33–39 weeks’ gestation.

**Methods:**

This retrospective study evaluated 5298 singleton pregnancies that had involved three ultrasound examinations at 21^+0^–27^+6^, 28^+0^–32^+6^, and 33^+0^–39^+6^ weeks. All ultrasound data were transformed to MoM values (multiple of the median). Multivariate logistic regression was used to analyze the correlation between SGA status and various variables (ultrasound data and MF) during pregnancy to build the SGAp model. EFW was calculated according to the Hadlock formula at 33–39 weeks of gestation. The predictive performance of SGAp, AC MoM value at 33^+0^–39^+6^ weeks (AC-M), EFW MoM value (EFW-M), EFW-M plus MF, AC value at 33^+0^–39^+6^ weeks (AC), AC growth velocity, EFW, and EFW plus MF was evaluated using ROC curves. The detection rate (DR) of SGA neonate with SGAp, AC-M, EFW-M, and EFW-M plus MF at false positive rate (FPR) of 5% and 10%, and the FPR at DR of 85%, 90%, and 95% were observed.

**Results:**

The AUCs of SGAp, AC-M, EFW-M, EFW-M plus MF, AC, AC growth velocity, EFW, and EFW plus MF for SGA neonates screening were 0.933 (95%CI: 0.916–0.950), 0.906 (95%CI: 0.887–0.925), 0.920 (95%CI: 0.903–0.936), 0.925 (95%CI: 0.909–0.941), 0.818 (95%CI: 0.791–0.845), 0.786 (95%CI: 0.752–0.821), 0.810 (95%CI: 0.782–0.838), and 0.834 (95%CI: 0.807–0.860), respectively. The screening efficiency of SGAp, AC-M, EFW-M, and EFW-M plus MF are significantly higher than AC, AC growth velocity, EFW, and EFW plus MF. The DR of SGAp, AC-M, EFW-M, and EFW-M plus MF for SGA neonates were 80.4%, 69.6%, 73.8% and 74.3% at 10% FPR. The AUCs of SGAp, AC-M, EFW-M, and EFW-M plus MF 0.950 (95%CI: 0.932–0.967), 0.929 (95%CI: 0.909–0.948), 0.938 (95%CI: 0.921–0.956) and 0.941 (95%CI: 0.924–0.957), respectively for screening SGA neonates delivered within 2 weeks after the assessment. The DR for these births increased to 85.8%, 75.8%, 80.0%, and 82.5%, respectively.

**Conclusion:**

The rational use of ultrasound data can significantly improve the prediction of SGA statuses.

## Introduction

Small-for-gestational-age (SGA) status is defined by birth weight below the 10^th^ percentile of the mean weight for the same gestational age (GA) or two standard deviations below the mean weight for the same GA. The SGA status is associated with more risk factors and more complications. SGA is a crucial predictive index of surgical intervention for congenital heart disease (CHD), and the coexistence of SGA and CHD is more likely to lead to death than either alone [1, 2]. One study published in 1997 reported that the incidence of suspected fetal asphyxia was threefold (6%-8%) higher in SGA fetuses than in non-SGA fetuses [3]. The perinatal mortality rate of SGA fetuses was high, and survivors showed adverse neurocognitive development leading to non-severe neurological dysfunction [4, 5].

Several approaches have been used to predict SGA neonates during pregnancy, some of which are summarized below. (1) Estimated fetal weight (EFW) measurements [6–8]: Most EFW formulas show a strong correlation between the predicted weight and actual birth weight (*r* > 0.9, 19/21 formulas) [6]. The area under the curve (AUC) for predictions based on EFW measurements in the mid-trimester or third-trimester ultrasound was 0.69 ~ 0.72 and 0.79, respectively [9, 10]. Ciobanu et al. showed that the AUC of EFW measurements was 0.883 at 35 and 36 weeks of gestation, while the AUC for births that occurred within two weeks of the evaluation could be as high as 0.933 [11]. (2) Ultrasound data [9, 11–13]: The AUC of the abdominal circumference (AC) growth velocity between 20 and 36 weeks was 0.808, while the AUC for births within two weeks of the evaluation was 0.884 [11]. However, many ultrasound data alone showed poor performance for predicting neonatal SGA [9, 12, 13]. (3) Biomarker evaluations: Biomarkers alone were not perfect for neonatal SGA prediction [14–16]. But Biomarkers and EFW in conjunction with maternal factors (MF) show good predictive value [16].

Therefore, this study aims to improve the detection rate (DR) of neonatal SGA screening by constructiong a SGA screening model (SGAp) through multivariate logistic regression modeling without increasing the cost of pregnancy examination.

## Methods

### Study design overview

**Fig. 1:**
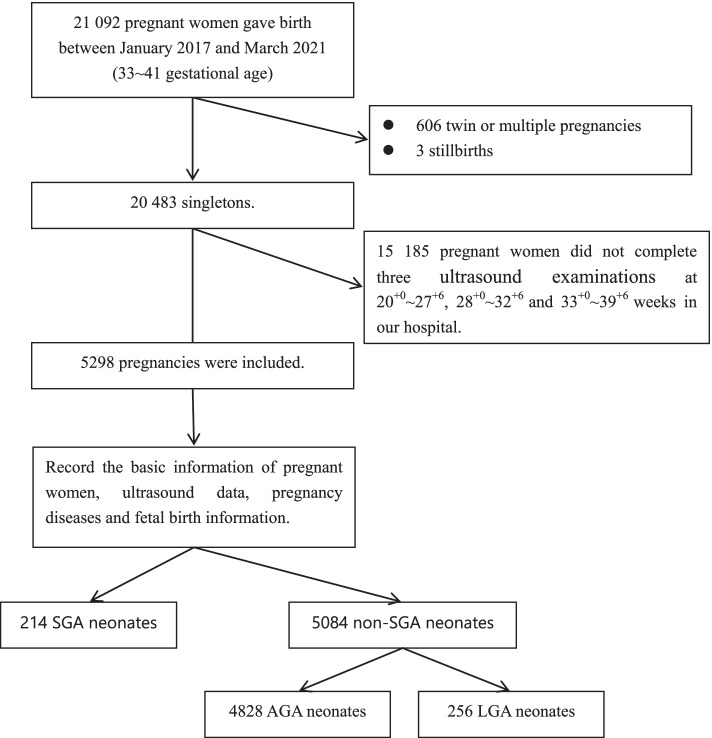
Flowchart of the study population

This was a retrospective study. A total of 21 092 pregnant women gave birth in Taizhou hospital from January 2017 to March 2021. Figure 1 outlines the data collection process and 5 298 pregnant women were included in this study. The inclusion criteria were single live births at 33 to 41 weeks’ gestation, and a history of at least three ultrasonographic examinations at 21^+0^–27^+6^, 28^+0^–32^+6^, and 33^+0^–39^+6^ weeks at our hospital. Considering the errors caused by different hospitals with different doctors and instruments, the reports form different hospitals were excluded. Maternal characteristics, diseases during the pregnancy, ultrasound data, and delivery information were recorded when the pregnant women came to our hospital for delivery.

The GA of the fetus was determined by the last menstrual period and the crown-to-rump length (CRL) of the fetuses at 11–14 weeks [17]. When the GAs determined by both methods were less than 1 week apart, the last menstrual period was chosen to determine the GA. However, when the GAs determined by both methods were more than 1 week apart, the GA based on ultrasound measurements was included in the analyses. All ultrasound examinations were performed by examiners with a certificate from a medical practitioner. In China, pregnant women younger than 35 weeks were required to participate in the first- and second-trimester screening or NIPT screening, and older and high-risk pregnant women were required to undergo amniocentesis to eliminate major fetal chromosome diseases. When pregnant women from 33 to 40 weeks of gestation came to our hospital for ultrasound appointment, we introduced the intention of this study to them and sign an informed consent form. All pregnant women, admitted to our hospital for delivery, were informed that all information about their pregnancy might be used anonymously in the future and signed informed consent. The study was approved by Taizhou Ethics Committee.

### Sample size calculation

The previously published report showed expected sensitivity towards EFW for detecting neonatal SGA to be 60.6% and specificity 87.6%, whereas for AC this rate was 64.4% and 83.5%, respectively, between 33^+0^ ~ 35^+6^ gestational weeks [18]. The sample size was calculated by software according to the above rates and a usable sample size of 88 patients was required in the anomalies group, with an allowable error of 0.1 in a two-tailed test with *p* < 0.05.

### Maternal characteristics

All pregnant women were Chinese. Maternal age, height, weight, body mass index (BMI), pregnancy history (the number of pregnancy, number of production, scarred uterus), and disease history during pregnancy were recorded.

Hypertension, diabetes, pre-eclampsia, vaginal inflammation (such as bacterial vaginosis, candida vaginosis, streptococcus lactis vaginitis, trichomonas vaginitis), viral infection (HBV, syphilis, HPV, etc.), intrahepatic cholestasis, thyroid dysfunction, abnormal placental shape, placental hypofunction, anemia or pelvic adhesionism during pregnancy might affect the health and development of the fetuses. As we all knew, pre-eclampsia was a type of hypertensive disorder of pregnancy that could lead to conditions such as fetal growth restriction and placental abruption. Gestational diabetes could cause fetal overgrowth. Vaginal inflammation might lead to infection, causing pelvic inflammatory disease and intrauterine infection. placental hypofunction (placental aging) might lead to hypoxia of the fetuses and even to the arrest of placental development.

The delivery process was also well-documented, and the mode of delivery, fetal distress, vaginal laceration, and the use of low forceps was recorded.

### Neonatal characteristics

Neonatal sex, weight, GA at birth, and neonatal asphyxia were recorded. According to the latest Chinese standards [19], a fetus weighing less than the 10^th^ percentile or more than 90^th^ percentile of the GA (≤ 36 weeks), and weighing less than 2500 g or more than 4000 g after term is considered a SGA neonate or large for GA (LGA) neonate, respectively. Neonates that do not meet the criteria for SGA and LGA statuses are called appropriate for gestational age (AGA) neonates.

### Ultrasonic data collection

All pregnant women had undergone ultrasound examinations at 20^+0^–27^+6^ weeks, 28^+0^–32^+6^ weeks, and 33^+0^–39^+6^ weeks. Head circumference (HC), abdominal circumference (AC), femur length (FL), biparietal diameter (BPD), occipitofrontal diameter (OFD), amniotic fluid index (AFI), placebtak thickness (PT), the ratio of systolic velocity / diastolic velocity of umbilical artery blood flow (S/D), pulsatile index of umbilical artery (PI), resistance index of umbilical artery blood flow (RI) were recorded. The 10^th^, 50^th^ and 90^th^ percentile values of all ultrasound data were counted according to gestational age.

### Transformed ultrasound data according to GA

All ultrasound data in this paper were transformed according to the local median of gestational ages, that was to say MoM values (multiple of the median). The reasons were as follows: At each GA, ultrasound data were not normally distributed. And each region had distinct differences due to climate and diet differences (e.g., northern and southern China). Therefore, MoM values were more appropriate than Z-Score when ultrasound data were transformed according to GA in this region.

### Estimated fetal weight (EFW)

The combination of AC, FL, BPD and HC yields a variety of formulas for calculating EFW [6]. According to the ultrasonic data from 33^+0^–39^+6^ weeks’ gestation, EFW was calculated respectively and finally converted into EFW MoM values (EFW-M) according to GA. ROC curve analysis showed that the formula created by Hadlock (1985) based on AC, FL and HC was the most suitable for this paper (Data not displayed).

### AC growth velocity

Previous article showed that AC growth velocity was better than EFW growth velocity for prediction of SGA neonate between 20 and 36 weeks [11]. The calculation method of AC growth velocity referred to this article.

### A SGA predictor (SGAp) model

Multivariate logistic regression was used to establish a SGAp model. Univariate logistic regression analysis was used to analyze the relationship between related variables and SGA neonates. The predicted SGA values were recalculated based on this model for all fetal conditions. The DR of SGA neonates was observed at 5% and 10% FPR. At the same time, The FPR of SGA was observed at the DR of 85%, 90% and 95%.

### Statistical analysis

Data were described in terms of medians/interquartile range (IQR) for continuous variables or numbers (n and %) for categorical variable. Mann–whitney U test, rank sum test or Chi-square test were used to compare differences between groups. Receiver operating characteristic (ROC) curves analysis was performed to evaluate the power of SGAp, AC MoM value at 33^+0^–39^+6^ weeks (AC-M), EFW-M, EFW-M plus MF, AC value at 33^+0^–39^+6^ weeks (AC), AC growth velocity, EFW, and EFW plus MF to discriminate SGA neonates. A *p* < 0.05 was considered to be significant.

## Results

### Patient characteristics

A total of 5 298 pregnancies with GAs of 33–41 weeks at birth were included in the study (Fig. 1 shows the inclusion process). The study population included 214 SGA (4.1%), 4828 AGA (91.1%), and 256 LGA (4.8%) neonates. The AGA and LGA neonates, collectively referred to as non-SGA neonates, served as the control group in this study. Basic information on the pregnant women, disease history during pregnancy, delivery, and newborn information are shown in Table 1. Weight, height, and BMI during pregnancy, proportion of boys, gestational week of delivery, and the proportion of streptococcal vaginitis in the women who delivered SGA neonates were significantly lower than those in women who delivered AGA and LGA neonates. On the other hand, the proportions of cesarean deliveries and patients with chronic hypertension and preeclampsia among women who delivered SGA neonates were significantly higher than those among women who delivered AGA and LGA neonates. Correlation analysis showed that SGA was correlated with maternal height, weight, BMI, fetal sex, and gestational disease history (streptococcus lactis vaginitis, gestational hypertension, preeclampsia, intrahepatic cholestasis during pregnancy), while LGA was associated with maternal age, height, weight, BMI, number of pregnancies, number of deliveries, baby sex, and gestational disease history (gestational diabetes, bacterial vaginitis, anemia).

**Table 1:** The characteristics of pregnant women, their pregnancies and neonates

**Characteristics**	**SGA**	**AGA**	**LGA**	P
	(*n* = 214)	(*n* = 4828)	(*n* = 256)	
**Maternal characteristics**				
Age (years)	29 (26–33)	30 (26–33)	31 (28–34)^**^	< 0.001
Predelivery weight (kg)	64 (59–72)^**^	68 (63–74)	74 (69–80)^**^	< 0.001
Height (cm)	158 (155–160)^**^	160 (157–163)	160 (158–164)^**^	< 0.001
BMI	25.8 (23.6–28.7)^**^	26.6 (24.8–28.9)	28.8 (26.6–30.7)^**^	< 0.001
The number of pregnancy	2 (1–3)	2 (1–3)	2 (1–4)^**^	0.014
The number of delivery	0 (0–1)	0 (0–1)	1 (0–1)^*^	0.004
**Newborn information**				
Baby gender				
Boy	85 (39.7%)^**^	2520 (52.2%)	183 (71.5%)^**^	< 0.001
Girl	129 (60.3%)	2308 (47.8%)	73 (28.5%)	
Gestational age at birth (weeks)	37 (37–38)^**^	38 (37–39)	39 (39–40)^**^	< 0.001
Premature infant (33–36 weeks)	45 (21.0%)^**^	418 (8.7%)	1 (0.4%)^**^	< 0.001
The birth weight of the baby	2355 (2195–2440)^**^	3250 (2990–3510)	4160 (4070–4300)^**^	< 0.001
Neonatal asphyxia	0 (0%)	15 (0.3%)	1 (0.4%)	0.921
**The delivery information**				
Scarred uterus	27 (12.6%)	756 (15.7%)	47 (18.4%)	0.233
Caesarean section	90 (42.1%)^**^	1362 (28.2%)	95 (37.1%)^**^	< 0.001
Colpoperineal laceration	71 (33.1%)^**^	2449 (50.8%)	102 (39.9%)^**^	< 0.001
Grade I vaginal laceration	60 (28.0%)^*^	1760 (36.5%)	58 (22.7%)^**^	< 0.001
Grade II vaginal laceration	11 (5.1%)^**^	689 (14.3%)	44 (17.2%)	< 0.001
Fetal distress in uterus	29 (13.6%)	554 (11.5%)	26 (10.2%)	0.511
Low forceps delivery	5 (2.3%)	249 (5.2%)	18 (7.0%)	0.069
**Diseases of pregnancy**				
vaginal inflammation	25 (11.7%)	559 (11.6%)	36 (14.1%)	0.484
Bacterial vaginosis	6 (2.8%)	111 (2.3%)	15 (5.9%)^**^	< 0.001
Candida vaginosis	20 (9.3%)	310 (6.4%)	18 (7.0%)	0.229
Streptococcus lactis vaginitis	2 (0.9%)^*^	201 (4.2%)	14 (5.5%)	0.035
Trichomonas vaginitis	1 (0.5%)	6 (0.1%)	1 (0.4%)	0.269
Gestational diabetes mellitus	46 (21.5%)	1088 (22.5%)	86 (33.6%)^**^	< 0.001
Hypertension	19 (8.9%)^**^	189 (3.9%)	8 (3.1%)	< 0.001
Pregnancy hypertension	12 (5.6%)	168 (3.5%)	6 (2.3%)	0.148
Chronic hypertension	7 (3.3%)^**^	21 (0.4%)	2 (0.8%)	< 0.001
Placental hypofunction	30 (14.0%)	554 (11.5%)	22 (8.6%)	0.177
Preeclampsia	29 (13.6%)^**^	118 (2.4%)	7 (2.7%)	< 0.001
Mild preeclampsia	12 (5.6%)^**^	81 (1.7%)	7 (2.7%)	< 0.001
Serious preeclampsia	17 (7.9%)^**^	37 (0.8%)	0 (0%)	< 0.001
Intrahepatic cholestasis during pregnancy	8 (3.7%)	79 (1.6%)	2 (0.8%)	0.076
Thyroid dysfunction	11 (5.1%)	411 (8.5%)	23 (9%)	0.368
Virus infection				
HBV	5 (2.3%)	111 (2.3%)	6 (2.3%)	0.998
Syphilis	1 (0.5%)	26 (0.5%)	1 (0.4%)	0.943
HPV	0 (0%)	12 (0.2%)	1 (0.4%)	0.688
Abnormal shape of placenta	19 (8.9%)	343 (7.1%)	18 (7.0%)	0.678
Pelvic adhesion	3 (1.4%)	97 (2.0%)	6 (2.3%)	0.761
Anemia	18 (8.4%)	562 (11.6%)	40 (15.6%)	0.048

Data are given as n (%) or median (interquartile range). *P < 0.05, **P < 0.001

### Ultrasound data at different gestational weeks

All pregnant women had undergone ultrasound examinations at 20^+0^–27^+6^ weeks, 28^+0^–32^+6^ weeks, and 33^+0^–39^+6^ weeks. In China, the most accurate detection time for abnormal ultrasound findings is 23–25 weeks, so a large number of people choose to undergo ultrasound at 24 weeks’ gestation. All data were grouped according to GA and described as median, 10^th^, and 90^th^ percentiles (Table 2). After all data were MoM value-transformed, rank-sum test analysis showed that the BPD, OFD, HC, FL, AC, and AFI of SGA fetuses were significantly lower than those of AGA and LGA fetuses at 20^+0^–27^+6^, 28^+0^–32^+6^, and 33^+0^–39^+6^ weeks’ gestation (Table 3).

**Table 2:** the 10^th^ percentile, 50^th^ percentile and 90^th^ percentile values of BPD, OFD, HC, FL, AC, AFI, S/D, PI, RI and PT for gestational age

GA	N	BPD			OFD			HC			FL			AC		
		10^th^	50^th^	90^th^	10^th^	50^th^	90^th^	10^th^	50^th^	90^th^	10^th^	50^th^	90^th^	10^th^	50^th^	90^th^
21^+0^ ~ 21^+6^	11	48	52	55	62	66	69	177	190	205	33	37	38	158	167	179
22^+0^ ~ 22^+6^	56	53	55	59	67	71	75	196	207	218	37	40	42	171	183	193
23^+0^ ~ 23^+6^	887	54	59	63	70	74	79	205	218	230	39	42	45	178	192	206
24^+0^ ~ 24^+6^	3549	57	60	64	72	76	80	213	223	233	41	43	46	187	198	209
25^+0^ ~ 25^+6^	684	59	62	66	75	79	83	221	231	242	43	45	48	194	206	217
26^+0^ ~ 26^+6^	80	61	65	68	77	82	86	229	240	252	44	47	50	197	214	224
27^+0^ ~ 27^+6^	31	58	68	73	74	87	91	218	254	264	42	50	52	195	226	240
28^+0^ ~ 28^+6^	686	69	72	76	87	91	95	255	265	277	51	53	56	230	242	255
29^+0^ ~ 29^+6^	1200	72	75	79	90	94	98	264	275	287	53	55	58	241	253	267
30^+0^ ~ 30^+6^	1708	74	77	81	92	96	101	272	282	293	55	57	60	250	262	275
31^+0^ ~ 31^+6^	915	76	80	83	95	99	104	279	289	301	57	59	62	259	273	285
32^+0^ ~ 32^+6^	789	78	82	86	97	102	106	286	297	309	59	61	63	267	282	295
33^+0^ ~ 33^+6^	589	80	84	88	100	104	109	292	304	317	60	63	66	278	293	307
34^+0^ ~ 34^+6^	1333	82	86	90	102	106	110	300	310	321	63	65	68	288	302	317
35^+0^ ~ 35^+6^	1386	85	88	92	104	108	112	306	316	328	64	67	69	297	312	327
36^+0^ ~ 36^+6^	1165	86	90	93	106	110	113	311	321	333	66	69	71	306	320	335
37^+0^ ~ 37^+6^	574	88	92	95	108	112	115	316	327	338	67	70	72	314	330	345
38^+0^ ~ 38^+6^	206	88	92	96	108	112	116	318	329	342	68	71	74	320	334	351
39^+0^ ~ 39^+6^	45	90	94	98	109	114	118	322	336	347	69	72	74	327	342	358
GA	N	AFI			S/D			PI			RI			PT		
		10^th^	50^th^	90^th^	10^th^	50^th^	90^th^	10^th^	50^th^	90^th^	10^th^	50^th^	90^th^	10^th^	50^th^	90^th^
21^+0^ ~ 21^+6^	11	95	116	127	2.95	3.11	3.80	1.03	1.11	1.29	0.66	0.68	0.74	21	22	25
22^+0^ ~ 22^+6^	56	94	119	141	2.46	2.96	3.59	0.90	1.08	1.26	0.60	0.67	0.72	21	24	28
23^+0^ ~ 23^+6^	887	92	119	157	2.38	3.05	3.84	0.85	1.08	1.28	0.58	0.67	0.74	20	25	31
24^+0^ ~ 24^+6^	3549	96	119	147	2.46	3.00	3.65	0.88	1.06	1.24	0.59	0.67	0.73	22	26	30
25^+0^ ~ 25^+6^	684	96	119	148	2.46	2.96	3.57	0.88	1.05	1.22	0.59	0.66	0.72	22	26	31
26^+0^ ~ 26^+6^	80	95	117	149	2.3	2.84	3.50	0.81	1.02	1.18	0.57	0.65	0.71	23	27	32
27^+0^ ~ 27^+6^	31	96	110	134	2.38	2.69	3.30	0.86	0.99	1.14	0.58	0.63	0.70	24	28	31
28^+0^ ~ 28^+6^	686	94	115	144	2.18	2.68	3.24	0.77	0.95	1.14	0.54	0.63	0.69	25	29	33
29^+0^ ~ 29^+6^	1200	92	112	143	2.14	2.62	3.10	0.75	0.94	1.09	0.53	0.62	0.68	26	30	34
30^+0^ ~ 30^+6^	1708	90	110	141	2.09	2.53	2.95	0.73	0.91	1.06	0.52	0.61	0.66	27	31	35
31^+0^ ~ 31^+6^	915	87	108	143	2.05	2.49	2.93	0.71	0.90	1.05	0.51	0.60	0.66	27	31	36
32^+0^ ~ 32^+6^	789	85	105	136	2.01	2.46	2.88	0.69	0.89	1.03	0.50	0.59	0.65	28	32	37
33^+0^ ~ 33^+6^	589	78	102	136	1.97	2.38	2.84	0.68	0.86	1.02	0.49	0.58	0.65	29	33	38
34^+0^ ~ 34^+6^	1333	80	101	134	1.92	2.32	2.79	0.65	0.84	1.02	0.48	0.57	0.64	29	33	38
35^+0^ ~ 35^+6^	1386	77	100	130	1.91	2.26	2.75	0.64	0.81	1.00	0.48	0.56	0.64	30	34	38
36^+0^ ~ 36^+6^	1165	78	99	130	1.88	2.24	2.73	0.63	0.81	0.99	0.47	0.55	0.63	30	34	39
37^+0^ ~ 37^+6^	574	76	98	135	1.82	2.18	2.66	0.60	0.78	0.97	0.45	0.54	0.62	31	35	39
38^+0^ ~ 38^+6^	206	71	99	135	1.84	2.16	2.61	0.61	0.78	0.98	0.46	0.54	0.62	31	35	38
39^+0^ ~ 39^+6^	45	63	90	125	1.82	2.11	2.52	0.61	0.77	0.96	0.45	0.53	0.61	31	35	39

*GA* Gestational age, *BPD* Biparietal diameter, *OFD* Occipitofrontal diameter, *HC* Head circumference, *FL* Femur length, *AC* Abdomen circumference, *AFI* Amniotic fluid index, Umbilical arterial flow, *S/D* Ratio of fetal umbilical artery systolic to diastolic blood pressure, *PI* Pulsatile index, *RI* Resistance index, *PT* The thickness of the placenta

**Table 3:** The BPD, OFD, HC, FL, AC, AFI, S/D, PI, RI MoM values were converted according to gestational age

GA	Parameters	SGA	AGA	LGA	*P*
		(*n* = 214)	(*n* = 4828)	(*n* = 256)	
21^+0^ ~ 27^+6^	BPD	0.98 (0.95–1.00)^**^	1.00 (0.97–1.03)	1.02 (0.98–1.05)^**^	< 0.001
	OFD	0.97 (0.94–1.00)^**^	1.00 (0.97–1.03)	1.01 (0.99–1.04)^**^	< 0.001
	HC	0.98 (0.95–1.00)^**^	1.00 (0.98–1.02)	1.02 (0.99–1.04)^**^	< 0.001
	FL	0.98 (0.95–1.00)^**^	1.00 (0.98–1.04)	1.02 (1.00–1.05)^**^	< 0.001
	AC	0.98 (0.94–1.00)^**^	1.00 (0.97–1.03)	1.03 (1.00–1.06)^**^	< 0.001
	AFI	0.95 (0.84–1.06)^**^	1.00 (0.89–1.12)	1.03 (0.94–1.14)^**^	< 0.001
	S/D	1.01 (0.89–1.16)	1.00 (0.90–1.11)	0.97 (0.86–1.04)^**^	< 0.001
	PI	1.00 (0.90–1.11)	1.00 (0.91–1.09)	0.98 (0.88–1.05)^**^	< 0.001
	RI	1.00 (0.94–1.07)	1.00 (0.94–1.04)	0.99 (0.91–1.02)^**^	< 0.001
	PT	0.96 (0.88–1.04)^**^	1.00 (0.92–1.08)	1.04 (0.96–1.12)^**^	< 0.001
28^+0^ ~ 32^+6^	BPD	0.96 (0.94–0.99)^**^	1.00 (0.98–1.03)	1.01 (1.00–1.05)^**^	< 0.001
	OFD	0.97 (0.94–0.99)^**^	1.00 (0.98–1.02)	1.02 (0.99–1.04)^**^	< 0.001
	HC	0.96 (0.95–0.99)^**^	1.00 (0.98–1.02)	1.02 (1.00–1.04)^**^	< 0.001
	FL	0.97 (0.95–1.00)^**^	1.00 (0.98–1.03)	1.02 (1.00–1.05)^**^	< 0.001
	AC	0.95 (0.93–0.98)^**^	1.00 (0.98–1.02)	1.04 (1.01–1.06)^**^	< 0.001
	AFI	0.98 (0.82–1.12)^*^	1.00 (0.89–1.13)	1.07 (0.97–1.23)^**^	< 0.001
	S/D	1.05 (0.96–1.14)^**^	1.00 (0.90–1.09)	0.96 (0.87–1.06)^**^	< 0.001
	PI	1.05 (0.96–1.14)^**^	1.00 (0.89–1.09)	0.96 (0.86–1.05)^**^	< 0.001
	RI	1.03 (0.97–1.08)^**^	1.00 (0.93–1.05)	0.97 (0.90–1.03)^**^	< 0.001
	PT	0.97 (0.90–1.06)	1.00 (0.93–1.06)	1.03 (0.97–1.12)^**^	< 0.001
33^+0^ ~ 39^+6^	BPD	0.95 (0.93–0.98)^**^	1.00 (0.98–1.02)	1.02 (1.00–1.05)^**^	< 0.001
	OFD	0.96 (0.95–0.99)^**^	1.00 (0.98–1.02)	1.02 (1.00–1.04)^**^	< 0.001
	HC	0.96 (0.94–0.98)^**^	1.00 (0.98–1.02)	1.02 (1.01–1.04)^**^	< 0.001
	FL	0.96 (0.93–0.99)^**^	1.00 (0.98–1.02)	1.02 (1.00–1.03)^**^	< 0.001
	AC	0.93 (0.91–0.96)^**^	1.00 (0.98–1.02)	1.05 (1.03–1.07)^**^	< 0.001
	AFI	0.88 (0.77–1.01)^**^	1.00 (0.89–1.15)	1.11 (0.97–1.33)^**^	< 0.001
	S/D	1.05 (0.96–1.19)^**^	1.00 (0.91–1.11)	0.95 (0.86–1.06)^**^	< 0.001
	PI	1.05 (0.95–1.19)^**^	1.00 (0.88–1.12)	0.94 (0.82–1.05)^**^	< 0.001
	RI	1.04 (0.96–1.11)^**^	1.00 (0.91–1.07)	0.96 (0.87–1.04)^**^	< 0.001
	PT	0.97 (0.91–1.06)^**^	1.00 (0.94–1.06)	1.03 (1.00–1.11)^**^	< 0.001

Data are given as median (interquartile range). *GA* Gestational age, *BPD* Biparietal diameter, *OFD* Occipitofrontal diameter, *HC* Head circumference, *FL* Femur length, *AC* Abdomen circumference, *AFI* Amniotic fluid index, Umbilical arterial flow, *S/D* Ratio of fetal umbilical artery systolic to diastolic blood pressure, *PI* Pulsatile index, *RI* Resistance index, *PT* The thickness of the placenta. *P < 0.05, **P < 0.001

### Logistic regression modeling

Multivariate logistic regression modeling was conducted for all factors and ultrasound data after transformation. SGA neonates were represented by the dichotomous variable. It was found that the variables significantly correlated with the history of hypertension in pregnant women (X_1_, normal blood pressure = 0, gestational hypertension = 1, chronic hypertension = 2), preeclampsia (X_2_, no preeclampsia = 0, mild disease = 1, severe disease = 2), OFD MoM value at 21^+0^–27^+6^ weeks (OFD-M-[21, 27]) (X_3_), FL MoM value at 21^+0^–27^+6^ weeks (FL-M-[21, 27]) (X_4_), AC MoM value at 28^+0^–32^+6^ weeks (AC-M-[28, 32]) (X_5_), BPD MoM value at 33^+0^–39^+6^ weeks (BPD-M) (X_6_), FL MoM value at 33^+0^–39^+6^ weeks (FL-M) (X_7_), AC MoM value at 33^+0^–39^+6^ weeks (AC-M) (X_8_), AFI MoM value at 33^+0^–39^+6^ weeks (AFI-M) (X_9_). This model is called the SGA predictor (SGAp) (As shown in the Table 4). SGAp = -59.496–0.784X_1_-0.693X_2_-9.377X_3_ + 7.26X_4_ + 14.578X_5_ + 14.903X_6_ + 8.436X_7_ + 26.531X_8_ + 2.087X_9_.

**Table 4:** Multivariate Logistic regression model for SGA neonatal prediction

Characteristics		β	OR	95% CI	*P*
X_1_	Hypertension	-0.784	0.46	0.29–0.73	0.001
X_2_	Preeclampsia	-0.693	0.5	0.34–0.74	0.001
X_3_	OFD-M-[21, 27]	-9.377	0	0–0.01	< 0.001
X_4_	FL-M-[21, 27]	7.26	1422	13.11–1.54*10^5^	0.002
X_5_	AC-M-[28, 32]	14.578	2.14*10^6^	1.11*10^4^–4.16*10^8^	< 0.001
X_6_	BPD-M	14.903	2.97*10^6^	1.04*10^4^–8.44*10^8^	< 0.001
X_7_	FL-M	8.436	4609	11.24–1.89*10^6^	0.006
X_8_	AC-M	26.531	3.33*10^11^	6.35*10^8^–1.75*10^14^	< 0.001
X_9_	AFI-M	2.087	8.06	3.44–18.91	< 0.001
Constant		-59.496	0		< 0.001

OFD-M-[21, 27], OFD MoM value at 21^+0^ ~ 27^+6^ weeks; FL-M-[21, 27], FL MoM value at 21^+0^ ~ 27^+6^ weeks; AC-M-[28, 32], AC MoM value at 28^+0^ ~ 32^+6^ weeks; BPD-M, BPD MoM value at 33^+0^ ~ 39^+6^ weeks; FL-M, FL MoM value at 33^+0^ ~ 39^+6^ weeks; AC-M, AC MoM value at 33^+0^ ~ 39^+6^ weeks; AFI-M, AFI MoM value at 33^+0^ ~ 39^+6^ weeks

Univariate logistic regression results also showed that these indexes were closely related to SGA neonates (Table 5). After MoM transformation, the ultrasound data were all between 0 and 2, which were close to the values of hypertension and eclampsia. Univariate logistic regression results illustrated that OFD-M-[21, 27], FL-M-[21, 27], AC-M-[28, 32], BPD-M, FL-M, AC-M had high risk factors for SGA (Table 5).

**Table 5:** Univariable Logistic regression for SGA neonatal prediction

Characteristics	OR	95% CI	*P*
Hypertension	0.43	0.30–0.62	< 0.001
Preeclampsia	0.28	0.22–0.37	< 0.001
OFD-M-[21, 27]	1.92*10^6^	5.12*10^4^–7.21*10^7^	< 0.001
FL-M-[21, 27]	2.95*10^5^	1.13*10^7^–7.68*10^9^	< 0.001
AC-M-[28, 32]	1.14*10^15^	2.03*10^13^–6.42*10^16^	< 0.001
BPD-M	5.73*10^16^	6.96*10^14^–4.72*10^18^	< 0.001
FL-M	6.43*10^15^	1.06*10^14^–3.90*10^17^	< 0.001
AC-M	2.49*10^20^	2.43*10^18^–2.55*10^22^	< 0.001
AFI-M	26.67	13.49–52.72	< 0.001

OFD-M-[21, 27], OFD MoM value at 21^+0^ ~ 27^+6^ weeks; FL-M-[21, 27], FL MoM value at 21^+0^ ~ 27^+6^ weeks; AC-M-[28, 32], AC MoM value at 28^+0^ ~ 32^+6^ weeks; BPD-M, BPD MoM value at 33^+0^ ~ 39^+6^ weeks; FL-M, FL MoM value at 33^+0^ ~ 39^+6^ weeks; AC-M, AC MoM value at 33^+0^ ~ 39^+6^ weeks; AFI-M, AFI MoM value at 33^+0^ ~ 39^+6^ weeks

### Prediction of SGA neonate by SGAp

**Fig. 2:**
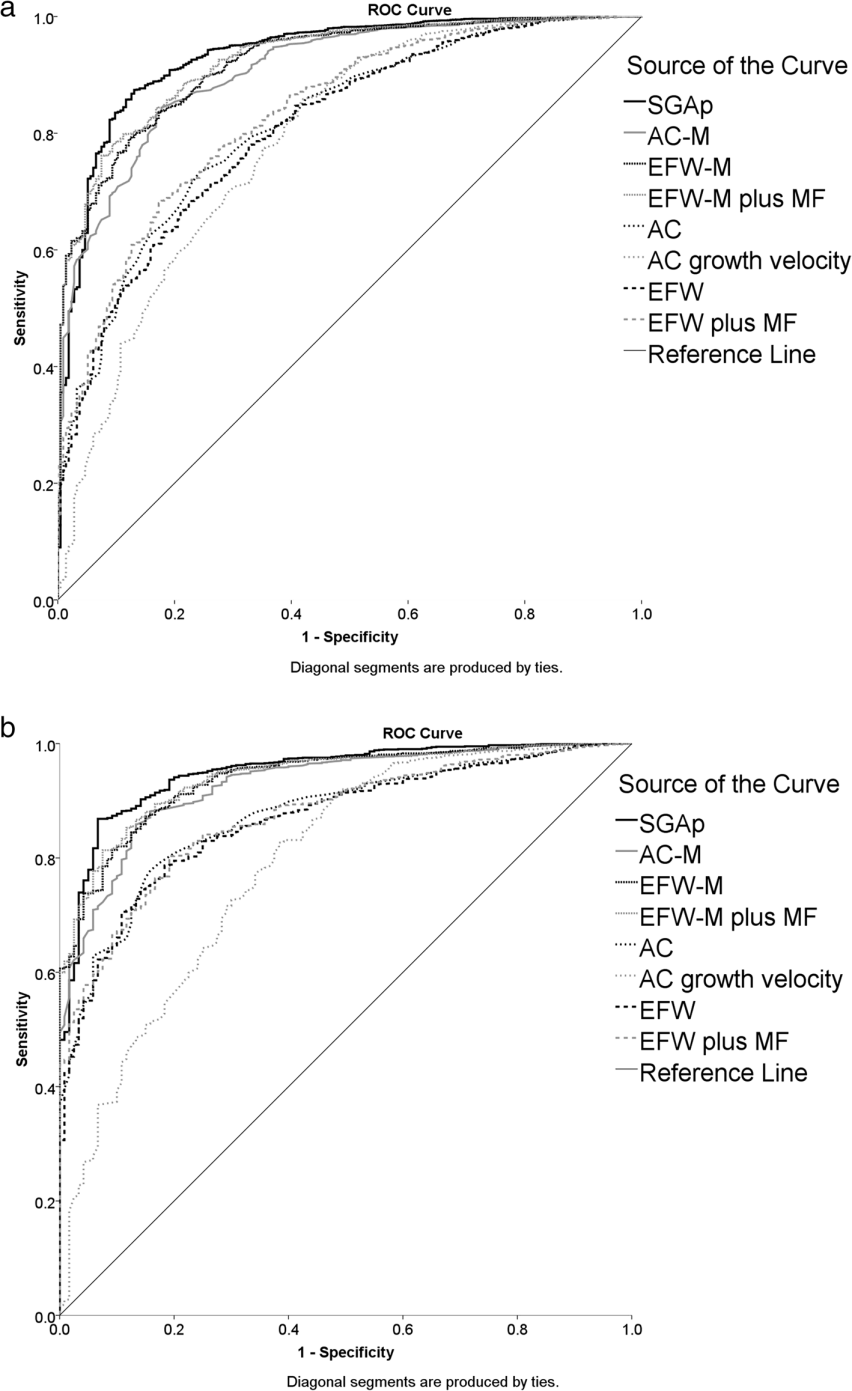
ROC curve analysis of SGAp, AC-M, EFW-M, EFW-M plus MF, AC, AC growth velocity, EFW, and EFW plus MF in prediction of SGA neonates delivered at any time (**a**) or within two weeks (**b**) after the assessment. AC-M, AC MoM value at 33^+0^ ~ 39^+6^ weeks; EFW-M, EFW MoM value at 33^+0^ ~ 39^+6^ weeks; EFW-M plus MF, EFW-M plus maternal factors

The SGAp values, AC-M, EFW-M, and EFW-M plus MF of fetuses in the SGA group were significantly lower than those in the non-SGA group (Table 6). The SGAp values were significantly different between the SGA and non-SGA groups (Table 6). The AUCs of SGAp, AC-M, EFW-M, EFW-M plus MF, AC, AC growth velocity, EFW, and EFW plus MF are showed in Fig. 2. The screening efficiency of SGAp, AC-M, EFW-M, and EFW-M plus MF are significantly higher than AC, AC growth velocity, EFW, and EFW plus MF. The DR of SGAp, AC-M, EFW-M, and EFW-M plus MF for SGA neonate screening at 5% and 10% FPR are shown in Table 7. The corresponding FPR of these four indicators at 85%, 90%, and 95% DR are also shown in Table 8.

**Table 6:** The median of SGAp, AC-M, EFW-M and EFW-M plus maternal factors in SGA group and non-SGA group

Characteristic	SGA	non-SGA	*p*
	median (IQR)	median (IQR)	
SGA born at any stage (N)	214	5084	
SGAp	0.96 (-0.47–2.39)	5.14 (3.91–6.36)	< 0.001
AC-M	0.93 (0.91–0.96)	1.00 (0.98–1.03)	< 0.001
EFW-M	0.84 (0.79–0.90)	1.00 (0.95–1.06)	< 0.001
EFW-M plus MF	34.56 (33.42–36.12)	38.38 (37.19–39.56)	< 0.001
SGA born within 2 weeks (N)	120	1459	
SGAp	0.61 (-0.79–1.93)	5.08 (3.91–6.35)	< 0.001
AC-M	0.93 (0.91–0.96)	1.00 (0.98–1.03)	< 0.001
EFW-M	0.82 (0.78–0.89)	1.01 (0.95–1.06)	< 0.001
EFW-M plus MF	34.35 (33.40–35.68)	38.43 (37.28–39.62)	< 0.001

AC-M, AC MoM value at 33^+0^ ~ 39^+6^ weeks; EFW-M, EFW MoM value at 33^+0^ ~ 39^+6^ weeks; EFW-M plus MF, EFW-M plus maternal factors

**Table 7:** The DR of SGAp, AC-M, EFW-M, and EFW-M plus MF for SGA neonate screening at 5% and 10% FPR

Screening test	DR at 5% FPR (%)	DR at 10% FPR (%)
SGA born at any stage		
SGAp	70.6 (67.4–73.7)	80.4 (77.7–83.1)
AC-M	60.7 (57.4–64.1)	69.6 (66.5–72.8)
EFW-M	66.4 (63.1–69.6)	73.8 (70.8–76.8)
EFW-M plus MF	67.8 (64.6–71.0)	74.3 (71.3–77.3)
SGA born within 2 weeks		
SGAp	74.2 (70.2–78.2)	85.8 (82.7–89.0)
AC-M	66.7 (62.4–71.0)	75.8 (71.9–79.7)
EFW-M	70.8 (66.7–75.0)	80.0 (76.3–83.7)
EFW-M plus MF	72.5 (68.4–76.6)	82.5 (79.0–86.0)

AC-M, AC MoM value at 33^+0^ ~ 39^+6^ weeks; EFW-M, EFW MoM value at 33^+0^ ~ 39^+6^ weeks; EFW-M plus MF, EFW-M plus maternal factors

**Table 8:** The false positive rate necessary to achieve prediction of 85%, 90% and 95% SGA neonates

screening test	FPR for 85% DR (%)	FPR for 90% DR (%)	FPR for 95% DR (%)
SGA born at any stage			
SGAp	11.8 (11.3–12.3)	16.4 (15.9–17.0)	27.8 (27.1–28.4)
AC-M	22.0 (21.4–22.6)	29.9 (29.3–30.6)	38.7 (38.1–39.4)
EFW-M	19.3 (18.7–19.8)	24.4 (23.8–25.1)	33.1 (32.5–33.8)
EFW-M plus MF	17.2 (16.7–17.8)	20.9 (20.3–21.5)	31.1 (30.5–31.8)
SGA born within 2 weeks			
SGAp	9.4 (8.6–10.2)	12.4 (11.5–13.3)	22.0 (20.9–23.1)
AC-M	13.2 (12.3–14.1)	23.0 (21.9–24.1)	32.7 (31.5–33.9)
EFW-M	13.1 (12.2–14.0)	18.0 (17.0–19.0)	26.2 (25.0–27.3)
EFW-M plus MF	12.6 (11.7–13.5)	17.0 (16.0–18.0)	26.1 (25.0–27.3)

AC-M, AC MoM value at 33^+0^ ~ 39^+6^ weeks; EFW-M, EFW MoM value at 33^+0^ ~ 39^+6^ weeks; EFW-M plus MF, EFW-M plus maternal factors

## Discussion

### Main findings

The present study confirmed that the SGAp, which was constructed using ultrasound data obtained in the second and third trimesters along with data for maternal history of hypertension and preeclampsia, showed better screening ability than EFW. The AUCs of SGAp, AC-M, EFW-M, EFW-M plus MF, AC, AC growth velocity, EFW, and EFW plus MF for SGA neonate screening were 0.933 (95%CI: 0.916–0.950), 0.906 (95%CI: 0.887–0.925), 0.920 (95%CI: 0.903–0.936), 0.925 (95%CI: 0.909–0.941), 0.818 (95%CI: 0.791–0.845), 0.786 (95%CI: 0.752–0.821), 0.810 (95%CI: 0.782–0.838), and 0.834 (95%CI: 0.807–0.860), respectively. However, all eight measures (SGAp: 0.950, 95%CI: 0.932–0.967; AC-M: 0.929, 95%CI: 0.909–0.948; EFW-M: 0.938, 95%CI: 0.921–0.956; EFW-M plus MF: 0.941, 95%CI: 0.924–0.957; AC: 0.874, 95%CI: 0.847–0.900; AC growth velocity: 0.791, 95%CI: 0.746–0.837; EFW: 0.866, 95%CI: 0.839–0.893; EFW plus MF: 0.873, 95%CI: 0.847–0.899) showed more effective screening performance if birth occurred within two weeks of the assessment. The SGA screening efficiency of data transformed by MoM value was significantly higher than that of data without MoM value transformation.

The DR of SGAp, AC-M, EFW-M, and EFW-M plus MF at 10% FPR were 85.8%, 75.8%, 80.0%, and 82.5%, respectively for screening SGA neonates delivered < 2 weeks after the assessment. The FPR of SGA screening by SGAp for 85%, 90%, and 95% DR were 9.4%, 12.4%, and 22.0%, respectively, in deliveries occurring < 2 weeks after the assessment.

The DR of SGAp, AC-M, EFW-M, and EFW-M plus MF for birth at any time were 80.4%, 69.6%, 73.8%, and 74.3%, respectively. The FPR of SGA fetal screening by SGAp for 85%, 90%, and 95% DR were 11.8%, 16.4%, and 27.8%, respectively.

### Strengths and limitations of the study

The strengths of this SGA neonatal screening study are as follows: First, based on the local median values for each GA, ultrasound data were MoM value-transformed, increasing their accuracy. Second, the study participants included pregnant women whose babies were born at 33–41 weeks (including preterm delivery). Third, the SGAp was based on prenatal ultrasound and maternal disease data, thereby ensuring better SGA screening than EFW.

The limitations of this study are as follows: First, this was a retrospective study. Of the 21092 pregnant women who gave birth in our hospital, 3/4 had been examined by ultrasound once or twice in our hospital, and most of them were tested in their local women's health care centers. Moreover, the ultrasound data were not incomplete in their local women's health care centers, resulting in a large amount of data loss. Second, the evaluations based on the SGAp model could only be performed after 33 weeks of gestation.

### Comparison with the findings of previous studies

We found that EFW-M and EFW-M plus MF assessments in the third trimester could predict 73.8% and 74.3%, respectively, of SGA neonates delivered at 33–41 weeks’ gestation at 10% FPR. Fadigas et al. used EFW Z-score (EFW-Z) and EFW-Z plus MF data at 35–37 weeks and reported that 63.1% and 66.0% of SGA neonates (< 10th percentile) were screened at 10% FPR [20]. Ciobanu et al. also used EFW-Z obtained at 35^+0^ to 36^+6^ weeks of gestation for screening SGA neonates (< 10^th^ percentile) and reported DR of 65.3% and 69.3% at 10% FPR for deliveries at ≥ 35 weeks’ gestation [21]. Bakalis et al. found that EFW-Z plus MF assessments at 30–34 weeks could predict 79.2% and 52.7% of SGA neonates with 10% FPR in deliveries occurring < 5 weeks and > 5 weeks after the assessments, respectively [16]. Overall, the screening effect of EFW-M was similar to that of the EFW-Z. However, it is easier to convert according to the GA.

This is the first study to combine ultrasound and MF data to construct an SGAp model. The SGAp model could screen 80.4% of SGA neonates at 10% FPR in deliveries at 33–41 weeks of gestation. For deliveries that occurred within two weeks of the evaluation, the DR increased up to 85.8%.

### Implications for clinical practice

In China, it is common for pregnant women to undergo ultrasound examinations five times during pregnancy: at 6–8 weeks, 12–14 weeks, 23–25 weeks, 29–31 weeks, and 34–36 weeks. In addition, some pregnant women in the third trimester may undergo ultrasound examinations every month or even at two-week intervals. Appropriate use of these ultrasound data is extremely important. AC and EFW growth velocities between 20 and 36 weeks of gestation cannot be used for effective screening^12^. Therefore, in this study, the most effective ultrasonic data across different stages were superimposed, and a logistic regression model was used to establish the SGAp model. The screening performance of the SGAp model was shown to be better than that of EFW. The three ultrasound data points used in this study were all obtained over a relatively large gestational range, ranging from 20 to 27 weeks, 28 to 32 weeks, and 33 to 39 weeks, improving the convenience of performing SGAp-based assessments in actual clinical practice.

## Conclusions

We aimed to evaluate the usefulness of the SGAp model for screening SGA neonates born at 33–41 weeks of gestation. The SGAp model could screen 80.4% of the SGA neonates at an FPR of 10%. The DR increased to 85.8% if the birth time was within two weeks of the assessment. Increasing the FPR further to 16.4% improved the SGA DR to 90% at any stage. Further research is needed to determine whether a larger sample size and more refined ultrasonic data can facilitate the establishment of a more accurate SGA screening tool.

## Data Availability

Data used or analyzed in this study are avaiable from the corresponding author on reasonable request.
